# Sex-Related Effects of Reproduction on Biomarkers of Oxidative Damage in Free-living Barn Swallows (*Hirundo rustica*)

**DOI:** 10.1371/journal.pone.0048955

**Published:** 2012-11-08

**Authors:** Diego Rubolini, Graziano Colombo, Roberto Ambrosini, Manuela Caprioli, Marco Clerici, Roberto Colombo, Isabella Dalle-Donne, Aldo Milzani, Andrea Romano, Maria Romano, Nicola Saino

**Affiliations:** 1 Dipartimento di Bioscienze, Università degli Studi di Milano, Milano, Italy; 2 Dipartimento di Biotecnologie e Bioscienze, Unversità degli Studi di Milano-Bicocca, Milano, Italy; 3 Dipartimento di Scienze biomediche per la salute, Università degli Studi di Milano, Milano, Italy; Institut Pluridisciplinaire Hubert Curien, France

## Abstract

According to life-history theory, the allocation of limiting resources to one trait has negative consequences for other traits requiring the same resource, resulting in trade-offs among life-history traits, such as reproduction and survival. In vertebrates, oxidative stress is increasingly being considered among the physiological mechanisms forming the currency of life-history trade-offs. In this study of the barn swallow (*Hirundo rustica*), we focus on the oxidative costs of reproduction, especially egg laying, by investigating the effects of breeding stage (pre- vs. post-laying) and progression of the season on three biomarkers of oxidative damage (OD) to plasma proteins, namely the concentration of malondialdehyde (MDA)-protein adducts and of protein thiol groups (PSH), and the protein carbonyl (PCO) content. Moreover, we investigated whether males and females differed in plasma OD levels, because the inherent sex differences in reproductive roles and physiology may originate sex-specific patterns of OD during breeding. We found that MDA-protein adduct levels were higher in the pre-laying than in the post-laying phase, that males had lower levels of MDA-modified proteins than females, and that the decline of MDA-protein adduct concentration between the pre- and the post-laying phase was more marked for females than males. In addition, MDA-protein adduct levels declined with sampling date, but only during the pre-laying phase. On the other hand, plasma PCO levels increased from the pre- to the post-laying phase in both sexes, and females had higher levels of PCO than males. PSH concentration was unaffected by breeding stage, sex or sampling date. On the whole, our findings indicate that biomarkers of protein oxidation closely track the short-term variation in breeding stage of both male and female barn swallows. Moreover, the higher protein OD levels observed among females compared to males suggest that egg laying entails oxidative costs, which might negatively affect female residual reproductive value.

## Introduction

Life-history theory posits that organisms trade investment in current reproduction against future reproductive prospects and survival [1]–[3]. Estimating the costs associated with different life-cycle activities, including reproduction, is therefore a crucial issue in studies investigating life-history trade-offs [4]–[7]. Indeed, the (negative) relationship between reproductive effort and survival or future reproduction has been widely documented in studies of diverse organisms, both via experimental and correlative approaches [8]–[13]. Most studies assume that life-history trade-offs are mainly regulated by resource based-costs, which occur when the internal resources available to an individual are limited and the energy and resource investment in one activity compete with the investment in another activity [4], [13], [14]. However, knowledge of the physiological, proximate, causes that form the currency of life-history trade-offs is still limited [5], [6], [15], [16]. In recent years, evidence is growing that oxidative stress plays an important role in mediating such trade-offs [17]–[20]. Oxidative stress results from an imbalance between the production of pro-oxidant substances, so called ‘reactive species’ (RS, sensu [21]), originating as by-products of oxidative metabolism and different physiological and pathophysiological functions, and the wide array of antioxidant defences and repair mechanisms that organisms have evolved to counteract the negative effects of RS. Indeed, RS can cause different degrees of oxidative damage (OD) to biological macromolecules and cells/tissues, which accumulates in the soma when antioxidant defence and repair mechanisms are overridden [17], [19], [20], [22]. Although it is becoming increasingly recognized that some RS play a key role in cell signalling [23], oxidative stress is known to impair physiological functions [22], may accelerate ageing, and cause higher susceptibility to environmental stress or pathogens [19], [24], thereby resulting in reduced fitness.

Reproduction is a highly energetically demanding activity, involving synthesis and transport of reproductive material (e.g. yolk and albumen deposition in oviparous species) and hypertrophy of plastic organs (mainly gonads), as well as peculiar behaviours such as mate guarding, food provisioning in altricial species, or lactation [25]–[28]. During reproduction, metabolic rates are normally higher than during other life cycle stages [28], [29], potentially leading to higher generation of pro-oxidants and thus higher levels of OD, unless antioxidant defences are sufficiently upregulated. Studies investigating oxidative stress in relation to reproduction have provided mixed evidence, perhaps because of the huge variation in methodologies to assess oxidative status, taxonomic focus, and sampling/experimental design [30]–[38]. For example, a study of captive zebra finches has shown a decrease in antioxidant defences following experimentally increased reproductive effort [32], suggesting that high reproductive effort may result in oxidative stress and decreased longevity. Other studies of birds have addressed the effects of reproductive activities on OD more directly, showing higher OD levels in individuals that were actively reproducing compared with those that were experimentally prevented from reproducing [36] or among females that were forced to increase egg laying effort [37]. On the other hand, among mammals, studies vary from showing a positive relationship between litter size and OD levels [33], to showing no relationships between current or past reproductive effort and OD biomarkers [34], or even lower levels of OD in females that were involved in reproductive activities compared to those that were prevented from reproducing [35]. The latter two studies suggest that either reproductive effort is less costly than expected in terms of OD, or that the antioxidant system of animals can efficiently cope with higher metabolic rate and intense reproduction-related stress [39], [40]. Moreover, fundamental sex differences in reproductive physiology, socio-sexual behaviour or parental investment may differently affect the production of RS and/or the functioning of the antioxidant system according to sex [30], [36], [41], [42]. For example, patterns of circulating hormones during reproduction differ markedly between the sexes [43], [44], and may affect oxidative status via several, non-mutually exclusive, physiological pathways [22].

Importantly, there are different pathways by which RS can cause OD, and levels of OD may vary according to stage in the life cycle and/or may be tissue-specific [19], [22]. Indeed, many validated biomarkers of OD to specific biomolecules exist [45]. Among these, protein carbonylation on the whole [46], [47] and, more specifically, the carbonylation derived from the covalent adduction of malondialdehyde (MDA), one of the main lipid peroxidation end-products [48], constitute the most widely adopted biomarkers of protein and lipid OD, respectively [45]. Protein carbonyl (PCO) groups (aldehydes and ketones) are chemically stable moieties that are introduced into proteins via disparate direct or indirect oxidative reactions [45]–[47]. Protein carbonylation is elicited by relatively severe oxidative stress and constitutes an irreversible type of OD [45]–[47], leading to a loss of protein structural and functional efficiency, with oxidised proteins being degraded by cells within hours or days [49], [50]. PCO content of tissues is considered the most general indicator of severe protein OD [46]. On the other hand, MDA, one of the most studied lipid electrophiles, is a major truncated oxidation product generated from oxidation of both free fatty acids and phospholipid esters during lipid peroxidation [45]; it can form stable adducts with proteins, whose concentration can be used as a proxy of lipid peroxidation [45], [51]. Levels of protein OD can also be evaluated by quantifying the circulating concentrations of protein thiol groups (PSH) [46]. Thiols show a high reaction rate with RS and are highly susceptible to oxidation [22]. A decrease in the plasma concentration of PSH is often correlated to conditions of oxidative stress [52], [53]. Moreover, reversible oxidation of PSH is involved in redox regulation of cell functions and gene expression [54], [55]. Clearly, quantifying different aspects of an organism’s redox state by measuring several OD biomarkers may allow an in-depth evaluation of the fitness consequences of oxidative stress [19], [20].

In this study, we investigated the short-term variation of plasma levels of MDA, PCO and PSH in a free-living migratory bird, the barn swallow (*Hirundo rustica*), in relation to stage in the reproductive cycle. Specifically, we aimed at investigating variation in plasma OD levels in birds sampled before egg laying, soon before the rapid yolk deposition (RYD) phase, and soon after egg laying, at the start of the incubation period (incubation does not start until clutch completion; [56]). The RYD phase, during which yolk is formed and rapidly deposited into follicles, begins up to 4–5 days before onset of egg laying in barn swallows and continues through clutch completion, until the last egg is ovulated, with the progressive maturation of follicles [57]. We sampled both female birds, that are involved in incubation [56], as well as males, that perform mate-guarding by actively following their mates during the fertile period until the termination of egg laying, but do not incubate eggs and do not provide food to their laying/incubating partners [56], [58]. Previous studies of field metabolic rates in breeding female barn swallows revealed that egg laying is not more costly than incubation or chick rearing [59], although the metabolic/hormonal machinery underlying the different reproductive phases differs largely [16], [60], possibly resulting in variation of oxidative stress biomarkers in relation to breeding stage. Moreover, inherent differences in reproductive physiology and behaviour between the sexes across egg laying, including differential variation in reproductive hormones (e.g. GnRH, sex hormones, and prolactin; [44]), may interact with redox state and generate sex-specific variation in OD levels between the pre- and post-laying phase.

## Materials and Methods

### Study Species and General Methods

The barn swallow is a small (15–20 g), semi-colonial, passerine bird feeding on aerial insects [56]. European populations are largely migratory, spending the boreal winter in sub-Saharan Africa [56]. The species breeds in colonies, mainly in rural buildings, usually in close association with livestock farming [61]. It breeds in April-July, laying up to three clutches of 3–7 eggs [56]. Females normally lay one egg per day, and incubation starts the day before that of clutch completion [56].

The study was carried out at 11 colonies located in the surroundings of Milano (N Italy) during spring-summer 2011. Adult barn swallows were captured (under permit by Regione Lombardia, Decreto n°2141, issued on March 9, 2011) with mist-nets inside rural buildings at regular intervals (ca. one capture session per month) between April and July, thus encompassing the entire period when first clutches are laid. Upon first capture, all birds were uniquely marked with coloured plastic bands and a combination of colour markings on the breast and belly to allow individual identification and assignment of all individuals to their nest via direct observations with binoculars. A blood sample (ca. 100 µl) was taken from the ulnar vein (see Ethics Statement below). Sex was unambiguously determined by checking the presence of a brood patch [62] (only females develop a brood patch; [56]). Laying date was estimated by inspecting all nests of the study colonies at least once per week, under the assumption that females lay one egg per day.

For the purpose of this study, we included data for first clutches only. We selected 71 individuals (38 females, 33 males) for which we could obtain accurate information on laying date (approximation ±1 d). We compared variation in OD biomarkers between stages in the breeding cycle (before or after laying; breeding stage hereafter). To this aim, as birds in the pre-laying period, we chose all the females that where captured and blood-sampled between 13 and 5 days before the onset of laying (mean time elapsed between sampling and start of laying: 9.53 (3.09 s.d.) days, *n* = 19), and all the males that were captured and blood-sampled between 13 and 5 days before their partner started laying (mean value: 8.07 (2.98 s.d.) days, *n* = 15). As birds in the post-laying period, we selected all the females that were captured and blood sampled between 4 and 9 days after onset of egg laying (mean value: 6.84 (1.68 s.d.) days, *n* = 19), and all the males that were first captured and blood sampled between 4 and 9 days after onset of egg laying by their partner (mean value: 7.00 (1.19 s.d.) days, *n* = 18). Males and females did not differ significantly in the mean number of days elapsed before laying during the pre-laying period (*t*_32_ = 1.39, *P* = 0.18), or after laying during the post-laying period (*t*_35_ = 0.32, *P* = 0.74). Post-laying females were sampled between 0 and 6 days after termination of egg laying (mean value 2.89 (1.79 s.d.) days). All individuals included in the analyses were sampled once (i.e. there are no repeated data for any individual in the dataset). Males and females belonged to the same breeding pair in 20 cases (i.e. there were 20 pairs of males and females that shared the same value of nest identity). Plasma samples were obtained by centrifugation and preserved at −80°C until biochemical analyses.

### Biomarkers of Plasma OD

#### Determination of MDA-protein adducts concentration

Plasma protein concentration was determined by Bradford assay. Concentration of MDA-protein adducts was determined using OxiSelect™ MDA Adduct ELISA Kit (Cell Biolabs, San Diego, CA, USA). A MDA-BSA standard curve (0–120 pmol/mg) and plasma samples (2–3 aliquots per individual, depending on sample availability) were processed according to manufacturer’s protocol. Briefly, samples were diluted to 10 µg/ml in 50 mM potassium phosphate buffer (PBS), pH 7.4, and added to 96-well protein binding plates. After overnight incubation at 4°C on an orbital shaker, the plate was washed twice with PBS, incubated 2 h at room temperature (RT) and washed three times before incubation with anti-MDA antibodies at 1∶1000 dilution (1 h, RT). The plate was then washed three times and incubated with horseradish peroxidase-conjugated secondary antibody at 1∶1000 dilution (1 h, RT). After five washes, RT-warmed substrate solution was added to each well. Colorimetric reaction was allowed to develop and stopped within 20 min. The plate was immediately read using Infinite F200 PRO TECAN. The mean value of the 2–3 readings for each individual was used in the analyses. Two outliers (values >3 s.d. of the mean) were excluded from all subsequent analyses, resulting in a sample size of 69 individuals. The intra- and inter-assay coefficients of variation were 5.91% and 16.39%, respectively. Repeatability, as estimated by the intraclass correlation coefficient (ICC) [63], was very high and statistically significant (ICC = 0.94, 95% c.l. 0.92–0.96, *n* = 69).

#### Determination of PSH concentration

Plasma protein samples (100 µg, 2 aliquots per individual) were mixed with three volumes of 100% acetone, and proteins were allowed to precipitate for 30 min at −20°C, followed by centrifugation at 10000 g for 10 min, at 4°C. Protein pellets were washed once with 1 ml of 70% acetone and centrifuged at 10000 g for 10 min, at 4°C. After dry vacuum, protein pellets were resuspendend in N-[6,7-(amino-4-methylcoumarin-3-acetamido)hexyl]-30-[20-pyridyldithio] propionamide (AMCA-HPDP; ThermoFisher Scientific, Rockford, IL, USA) solution (100 µM in 50 mM PBS, pH 7.4) at a molar ratio of 1∶6 in favour of AMCA-HPDP. Samples were incubated for 60 min at RT in the dark to ensure complete blocking of exposed sulphydryl groups. AMCA-HPDP labelled samples were precipitated and washed with acetone, as described above, and resuspended in PBS. The fluorescence-emission spectra of labelled plasma proteins (0.015 µg/µl in PBS) were determined at 25°C by using an excitation wavelength of 345 nm and scanning at emission wavelengths from 300 to 550 nm. Fluorescence data were collected with a Kontron SFM-25 spectrofluorometer, by using 10×10 mm acrylic cuvettes [64]. The mean value of the two aliquots for each individual was used in the analyses. Two outliers (values >3 s.d. of the mean) were excluded from all subsequent analyses, resulting in a sample size of 69 individuals. The intra-assay coefficient of variation was 3.83%, and repeatability was very high and statistically significant (ICC = 0.96, 95% c.l. 0.94–0.98, *n* = 69).

#### Quantification of the plasma PCO content

**Figure 1:**
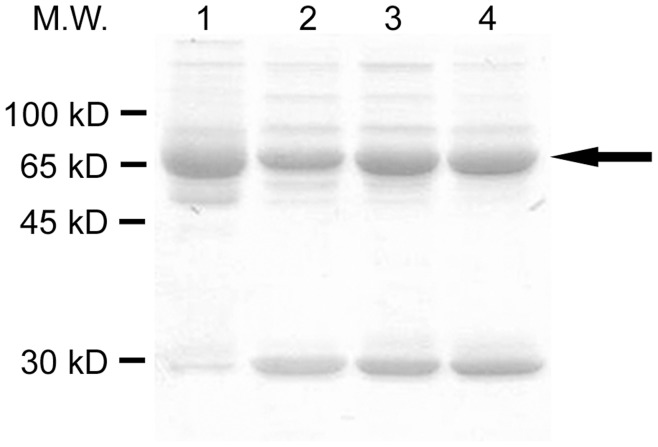
SDS-PAGE gel (10% w/v) representing human (lane 1) and swallow (lanes 2–4) plasma protein migration pattern after Coomassie Brilliant Blue staining. The black arrow indicates serum albumin. Molecular Weight Standards (M.W.) migration is shown on the left.

Total plasma proteins (2.5 µg, one single aliquot per individual due to sample limitation) were separated on 10% (w/v) reducing SDS–PAGE gels and electroblotted onto a polyvinylidene difluoride (PVDF) membrane. Figure 1 shows a comparative SDS-PAGE (10% gel) pattern of human (lane 1) and swallow (lanes 2–4) plasma proteins, with the arrow indicating serum albumin. After Western blot, membrane was incubated in 2 M HCl and afterwards in 2,4-dinitrophenylhydrazine (DNPH; 0.1 mg/ml in 2 M HCl) (Sigma-Aldrich, Milan, Italy) for 5 min each [65]. The membrane was then washed 3 times in 2 M HCl and 7 times in 100% methanol for 5 min each, followed by one wash in PBST [10 mM Na phosphate, pH 7.2, 0.9% (wt/vol) NaCl, 0.1% (vol/vol) Tween-20] and blocking for 1 h in 5% (wt/vol) nonfat dry milk in PBST. Carbonyl formation was probed by 2-h incubation with 5% milk/PBST containing anti-dinitrophenyl-KLH (anti-DNP) antibodies (1∶20000 dilution) (Molecular Probes, Eugene, OR, USA) [66]. After three washes with PBST for 5 min each, the membrane was incubated with a 1∶20000 dilution of the secondary antibody linked to horseradish peroxidase in 5% milk/PBST for 1 h. After washing 3 times with PBST for 5 min each, immunostained protein bands were visualized with enhanced chemiluminescence detection (GE Healthcare, Milan, Italy). Densitometric analysis was performed after scanning the chemiluminescence films by using ImageJ 1.40d [67]. PCO content was expressed in arbitrary units (a.u.). One outlier (value >3 s.d. of the mean) was excluded from all subsequent analyses, and there was no sufficient plasma for PCO determination in another individual, resulting in a sample size of 69 individuals.

### Statistical Analyses

**Table 1:** Linear models of oxidative damage biomarkers in relation to sex, breeding stage and sampling date.

	MDA-protein adducts			PSH			PCO		
	*F*	d.f.	*P*	*F*	d.f.	*P*	*F*	d.f.	*P*
Sex	0.06	62	0.80	0.66	65	0.42	25.37	65	<0.001^c^
Breeding stage	11.30	62	0.001	0.61	65	0.44	8.98	65	0.004^d^
Sampling date	10.55	62	<0.001	0.66	65	0.42	1.14	65	0.29
Sex×breeding stage	13.22	62	<0.001^a^	*0.58*	*62*	*0.45*	*1.82*	*62*	*0.18*
Sex×sampling date	1.40	62	0.24	*2.69*	*62*	*0.11*	*0.53*	*62*	*0.47*
Breeding stage×sampling date	9.22	62	0.004^b^	*2.42*	*62*	*0.12*	*2.72*	*62*	*0.11*
Sex×breeding stage×sampling date	*1.14*	*61*	*0.29*	*1.49*	*61*	*0.23*	*1.63*	*61*	*0.21*

Where appropriate, models were run while controlling for heterogeneity of variances between levels of the fixed factors (see Materials and Methods for details). Terms shown in italics were removed from the model (see Materials and Methods for details).

a : estimated mean values (s.e.): males, before laying = 1.81 (0.33), after laying = 0.74 (0.11); females, before laying = 3.62 (0.30), after laying = 0.81 (0.10).

b : estimated slopes (s.e.): before laying =  −0.06 (0.02), *t* =  −2.71, *P* = 0.009; after laying = 0.01 (0.01), *t* = 0.99, *P* = 0.33.

c : estimated mean values (s.e.): males = 1.43 (0.04); females = 1.77 (0.06).

d : estimated mean values (s.e.): before laying = 1.51 (0.05); after laying = 1.69 (0.05).

We relied on linear models to investigate variation in OD biomarkers in relation to sex, breeding stage, and sampling date (reflecting seasonal variation), as well as their two- and three-way interaction terms. Interaction terms of a given order were removed from the models in a single step if statistically non-significant (*P*>0.05) to reduce the chances of committing type I errors due to multiple testing [68]. Inclusion of farm and nest identity as random effects in mixed models (to account for possible non-independence of birds from the same breeding colony or the same breeding pair) did never improve model fit (AIC values of models with random effects were invariably higher or equal to those of models without random effects; details not shown for brevity), and the statistics for fixed effect terms were qualitatively unaltered by the inclusion of random effects. For simplicity, we will therefore present results of linear models rather than those of mixed models. However, exploratory analyses revealed a significant degree of heterogeneity in variances of MDA-protein adducts and PSH in relation to breeding stage, and in variances of PCO in relation to sex (details not shown for brevity). We therefore appropriately controlled for heterogeneity of variances by means of the *gls* function of the *nlme* library of R 2.8.1 (see [69]). We analysed the correlation between the different OD biomarkers by means of partial correlation tests, separately for each sex to account for any sex difference, while partialling out the possible confounding effects of breeding stage and sampling date. Similar partial correlation tests were carried out to assess the relationships between clutch size (as a proxy of primary reproductive effort) and OD biomarkers.

**Figure 2:**
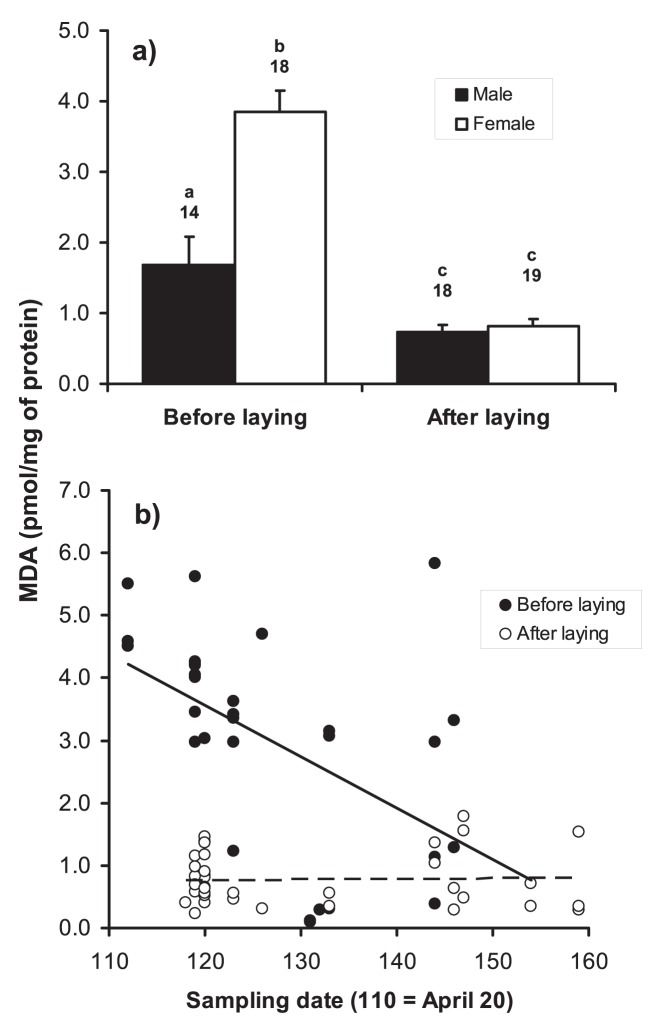
Concentration of MDA-protein adducts (pmol/mg of protein) in relation to a) sampling date and breeding stage and b) breeding stage and sex. a) Linear regressions are shown (continuous line = before laying; broken line = after laying); b) bars represent mean+s.e.; numbers above bars denote sample size, while letters denote statistically significant differences (*P*<0.05) between groups at *post-hoc* tests from the model presented in Table 1.

Sample sizes may differ slightly between analyses of different OD biomarkers because of missing values for some individuals, due to the exclusion of outliers or limitations in availability of tissue sample for biochemical analyses (see above).

**Figure 3:**
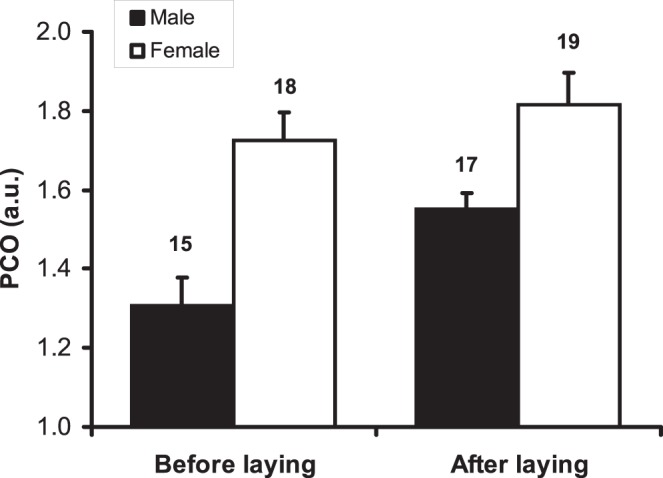
Plasma PCO content (mean+s.e.) in relation to sex and breeding stage. Numbers above bars denote sample size.

### Ethics Statement

Barn swallows were captured with mist-nets, extracted within 10 min of capture, kept safely in cloth bags, blood-sampled and released as soon as possible (usually within 1 h), following standard capture and handling techniques aimed at minimizing adverse effects. A small blood sample (ca. 100 µl) was collected by puncturing the ulnar vein with sterile needles, and the puncturing site accurately disinfected after sampling. After release, birds rapidly resumed their normal breeding activities, and no adverse effect of capture, handling and blood sampling was apparently observed. Both capture and blood sampling were authorized by Regione Lombardia (Decreto n°2141, issued on March 9, 2011). The study protocols were approved by the Ethics committee of the Department of Biosciences of the University of Milan.

## Results

### Sex- and Breeding Stage-dependent Variation in OD Biomarkers

The plasma concentration of MDA-protein adducts varied according to the combined effects of sex and breeding stage (Table 1, Fig. 2a). Specifically, levels of MDA-modified proteins markedly declined from the pre-laying to the post-laying period in both sexes, but the decline was more marked among females than males (Fig. 2a). In fact, before laying, females had higher levels of MDA-protein adducts than males, whereas no sex differences were detected after laying (Fig. 2a). Seasonal variation in concentration of MDA-protein adducts differed between the pre- and post-laying phases: MDA-protein adduct levels significantly declined with sampling date before laying, but did not covary with sampling date after laying (Table 1, Fig. 2b).

The concentration of PSH was unaffected by any of the considered factors or their interaction (Table 1), whereas we found a significantly higher PCO content in females compared to males (Table 1, Fig. 3), and a significant increase of PCO content from the pre- to the post-laying phase, which was similar in the two sexes (Table 1, Fig. 3).

### Covariation among OD Biomarkers

Partial correlation tests showed that the concentration of MDA-protein adducts did not significantly covary with PCO and PSH, neither in males (PSH, *r* = 0.03, d.f. = 28, *P* = 0.90; PCO, *r* = −0.21, d.f. = 27, *P* = 0.27) nor in females (PSH, *r* = 0.12, d.f. = 31, *P* = 0.52; PCO, *r* = −0.02, d.f. = 32, *P* = 0.91). On the other hand, while removing the confounding effects of breeding stage and sampling date, PSH and PCO values were significantly positively correlated in both sexes (males, *r* = 0.44, d.f. = 28, *P* = 0.014; females, *r* = 0.36, d.f. = 31, *P* = 0.040).

### Clutch size and OD Biomarkers

There was no statistically significant relationship between OD biomarkers and clutch size, neither in males (|*r*| <0.33, all *P*>0.08) nor in females (|*r*| <0.15, all *P*>0.38), while partialling out the effects of breeding stage and sampling date. The results were similar if tests were conducted separately for the pre- and post-laying period while partialling out the confounding effects of sex and sampling date (pre-laying: |*r*| <0.18, all *P*>0.33; post-laying: |*r*| <0.27, all *P*>0.12). Results were also similar if we used as measures of reproductive output the number of fledglings or the fledging success (ratio between the number of fledglings and clutch size) instead of clutch size (details not shown for brevity).

## Discussion

In this study, we investigated the short-term variation of three OD biomarkers between the pre- and the post-laying phase in male and female barn swallows. Plasma MDA-protein adduct concentration was significantly elevated in the pre- vs. the post-laying phase in both males and females. Moreover, MDA-protein adduct levels of females were significantly higher than those of males in the pre- but not in the post-laying phase, and declined with sampling date during the pre-laying phase in a similar fashion in the two sexes. Plasma PCO content increased from the pre- to the post-laying phase and was on average higher among females compared to males. The concentration of circulating PSH was unaffected by sex, sampling date, or breeding stage. There was no significant covariation between MDA-modified proteins and PSH or PCO, while there was a statistically significant, positive relationship between PSH and PCO. Finally, OD biomarkers were unrelated to reproductive investment.

Overall, females had higher levels of protein OD compared to males. This difference may be related to variation in metabolic rates between the sexes due to differential costs of gamete production, that are considerably larger for females than males [26], [70], possibly leading to higher RS production in females compared to males and causing higher levels of OD among the former. Moreover, sex differences in the hormonal cascade related to egg laying, including variation in sex steroids that are known to influence either directly or indirectly (via e.g. an effect on metabolic rates; [71]) an individual’s oxidative status [22], [72]–[74], may result in intersexual differences in OD. For example, Williams [16] suggested that the estrogen-dependent increase of circulating lipid-rich yolk precursors around egg laying might be associated with increased production of RS via lipid peroxidation, perhaps causing the higher MDA-protein adduct levels we observed in the pre- vs. the post-laying period in females compared to males in this study. Furthermore, two recent studies of captive bird species suggest that a raise in circulating estradiol levels can lead to increased oxidative stress [75], [76].

A higher OD among females compared to males supports the hypothesis that reproduction, especially around egg laying [16], could be more costly for females than for males in terms of oxidative stress. Indeed, some studies of birds suggest that the amount of energy and resources invested by females during reproduction, particularly across egg laying and incubation, is likely to be higher than that of males [70]. Previous studies revealed that egg laying is costly to females: mothers that were forced to lay extra eggs paid a survival cost [77]. Moreover, a higher degree of OD among females during reproduction, especially in terms of PCO content, that remains significantly higher in females than in males across egg laying, may contribute to explain the female-biased annual mortality rate of adult barn swallows ([58], but see [78]), a widespread pattern among avian species [79]. The finding of a higher degree of OD among female barn swallows, coupled with the observation that the sexes do not differ in terms of total plasma antioxidant capacity [80], suggest that oxidative stress may be causally involved in determining the lower survival of females compared to males. Females may in fact be unable to buffer the higher levels of OD to proteins and lipids that is generated around egg laying with higher levels of circulating antioxidants by upregulating their antioxidant system, and this may accelerate senescence and cause increased mortality compared to males.

The decrease of circulating MDA-modified proteins in the energy-demanding pre-laying phase (but not in the post-laying phase) according to progression of the season may indicate that the oxidative costs of reproduction are lower among late- vs. early-breeding individuals, irrespective of the investment in the current clutch, since there was no correlation between reproductive investment and OD levels. In the barn swallow, early reproduction is attained via early migration and arrival to the breeding grounds [81], and early migrating/arriving birds may experience highly unfavourable ecological conditions [58], that are relatively frequent during early spring at temperate latitudes. Such costs of early arrival are generally balanced by fitness payoffs, including a higher seasonal reproductive success, because of e.g. faster mate acquisition, or because of a longer breeding season, that may allow early reproducing individuals to lay more than one clutch [58], [82]. Secondly, seasonal differences in circulating MDA-modified proteins between early- and late-breeding birds may reflect differences in phenotype related to e.g. age or individual quality [81], [83]. A decline of MDA-modified protein levels with progression of the season thus either suggests that exposure to environmental stressors early in the season cause higher levels of OD, perhaps because of reduced antioxidant defences [84], or that early- and late-breeding birds differ in their susceptibility to oxidative stress.

Finally, we found a positive correlation between circulating plasma levels of PCO and PSH. This finding is puzzling as usually, when carbonylation becomes evident, exposed PSH have been already oxidized, and thus plasma PSH levels decline [85]. We might tentatively speculate that, in individuals experiencing high oxidative stress, causing significant levels of carbonylation, plasma antioxidant systems could be upregulated, which might in turn restore physiological PSH levels. Upregulation of antioxidant defence mechanisms could in fact directly protect PSH from reversible oxidation by RS. Alternatively, upregulation of antioxidant defences might reduce reversibly oxidized PSH. Under this scenario, higher plasma PSH levels might thus follow from high oxidative stress, thereby questioning the validity of plasma PSH as a biomarker of protein OD under natural conditions in avian species.

To conclude, our study highlighted that specific OD biomarkers, in particular MDA-protein adducts and PCO, are affected by the short-term variation in reproductive state across egg laying in a sex-specific way. These results add to the growing evidence that oxidative stress should be regarded among the physiological mechanisms contributing to the proximate costs of reproduction, possibly via the pleiotropic and sex-specific effects of reproductive hormones. Importantly, the higher levels of protein OD observed among females compared to males suggest that the oxidative costs of egg laying negatively affect female residual reproductive value, and may be involved in generating the female-biased mortality observed in the barn swallow.
